# Applications of SAR Interferometry in Earth and Environmental Science Research

**DOI:** 10.3390/s90301876

**Published:** 2009-03-13

**Authors:** Xiaobing Zhou, Ni-Bin Chang, Shusun Li

**Affiliations:** 1 Department of Geophysical Engineering, Montana Tech of The University of Montana, Butte, MT 59701, USA; E-Mail: xzhou@mtech.edu (X.Z.); 2 Department of Civil, Environmental, and Construction Engineering, University of Central Florida, 4000 Central Florida Blvd.; Orlando, FL 32816, USA; E-Mail: nchang@mail.ucf.edu (N.B.C.); 3 Geophysical Institute, University of Alaska Fairbanks, 903 Koyukuk Drive, P.O. Box 757320, Fairbanks, AK 99775-7320, USA; E-Mail: sli@gi.alaska.edu (S.L.)

**Keywords:** InSAR, phase, interferogram, deformation, remote sensing

## Abstract

This paper provides a review of the progress in regard to the InSAR remote sensing technique and its applications in earth and environmental sciences, especially in the past decade. Basic principles, factors, limits, InSAR sensors, available software packages for the generation of InSAR interferograms were summarized to support future applications. Emphasis was placed on the applications of InSAR in seismology, volcanology, land subsidence/uplift, landslide, glaciology, hydrology, and forestry sciences. It ends with a discussion of future research directions.

## 1. InSAR Overview of InSAR

### 1.1. Introduction

Interferometric synthetic aperture radar (InSAR) is a rapidly evolving remote sensing technology that directly measures the phase change between two phase measurements of the same ground pixel of the Earth’s surface. Two coherent synthetic aperture radar (SAR) phase images of the same portion of the Earth’s surface are required to form a phase difference image that is called an interferogram, in which a fringe pattern might appear. The two coherent SAR images used to form an interferogram can be acquired either from two antennas on the same space platform and separated perpendicularly to the flight direction (azimuth direction), a technique called single pass SAR interferometry (also called simultaneous interferometry), or from different passes of the same SAR antenna at different times, known as repeat-pass interferometry [1]. Any factor that can affect the phase of the backscattered radar signal can affect the fringe pattern and the number of fringes in the interferogram, and thus can potentially be measured by the InSAR technology. These measurements include surface displacements, land topography, land changes, land subsidence/uplift, water levels, soil moisture, snow accumulation, stem volume of forest, etc. Therefore, InSAR has found very broad applications in the field of earth and environmental sciences. Previous review articles on InSAR technology and its applications include [1–11]. These review articles summarized the technical development and applications results before 2001. The present review summarizes the most recent developments of InSAR remote sensing technology and its broad applications in earth and environmental systems, especially in the past decade. In the following discussion, we first review the fundamentals of InSAR briefly and then focus on InSAR applications in seismology, volcanology, land subsidence/lift, landslide, glaciology, hydrology, and forestry, respectively.

### 1.2. Phase Change–Basic Measurement of InSAR

**Figure 1.**
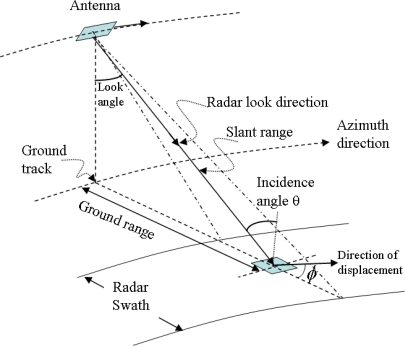
A geometric model for a SAR system. *Slant range* is the length between the antenna and ground pixel and *ground range* is the distance between the ground track and the ground pixel.

Radio detection and ranging (radar) is basically a tool for measuring the returned signal from the target and the distance between the target and the radar emitter (antenna). In contrast to real aperture radar systems, SAR makes use of the Doppler effect of the motion of platforms (satellite or aircraft) to increase the “aperture” and thus the resolution of the images. For imaging SAR, an electromagnetic (EM) wave pulse is emitted repeatedly in the cross-track direction (direction perpendicular to the moving direction of the antenna; Figure 1). The wave travels through atmosphere which contains electrically charged particles, aerosol and clouds, etc.; interacts with ground surface, and then part of the signal is scattered back to the receiving antenna.

To form an image from the echoes (returned signals) received from each emitted pulse, in the along-track direction (also called azimuth direction), the echoes are sorted by their round trip travel time, resulting in slant range resolution. At the same time, an airplane or satellite with the radar antenna moves forward and radar pulse is repeated. In SAR, the antenna must be designed to make azimuthal resolution as small as half the antenna length. This can be done if the antenna does not travel more than half of the *along-track* antenna length between the two successive pulses. It is also called the along-track resolution. The cross-track resolution and the along-track resolution determine the size of a ground pixel. Distance from the antenna to the ground is recorded in a rough form of slant range with additional information in the form of phase in the SAR image.

**Figure 2.**
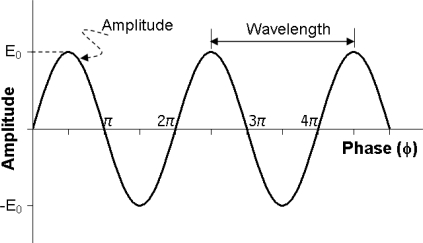
The relationship among amplitude, phase, and wavelength of a radar signal. The intensity of the radar signal is proportional to the squared amplitude.

The general expression for the electric field of a plane electromagnetic wave, which is a solution of the Maxwell equations, is:

(1) $$E=E_{0}\left(R,\;t\right)e^{i\left(k\cdot R-\omega t\right)}=E_{0}\left(R,\;t\right)e^{i\varphi }$$

where **E_0_**(*R,t*) is the amplitude of the EM pulse and the phase angle is:

(2) φ=k⋅R−ωt

where **k** = *k***ê** is the complex wave vector (**ê** is a unit vector in the propagation direction), *k* is the complex wave number, depending on dielectric constant. The wave propagates along space vector **R** (slant range). *ω* is the wave angular frequency and *t* the time. Thus, the echo received by the antenna of an imaging radar from a ground pixel is characterized by two quantities: *amplitude* and *phase,* as shown in Figure 2.

The digitized signal from a ground pixel is conveniently represented as a complex number, thus giving a complex image. Complex images are generated from the signal data received by each antenna. The amplitude of an image pixel represents the backscattering capability of the terrain of the corresponding ground pixel to send the incident energy back to the antenna. The pictorial representation of the amplitude of the EM pulses received by the antenna is called *amplitude image*. Backscattering and reflection are two different concepts. A very reflective calm water surface is very reflective but the least backscattering, so in a radar image, calm water surfaces correspond to the dark pixels.

The phase records the history of the signal from its emission to its return. The pictorial representation of the phase of the EM pulse is called *phase image*. In practice, however, an interferometric system does not measure the total pixel phase. Rather, it measures only the phase that remains after subtracting all full intervals present (module-2π). The phase image is thus called *wrapped*. Conversion from wrapped phase to continuous true phase is called *phase unwrapping*.

### 1.3. Factors That Impact the Phase of an Image Pixel

An *image pixel* is a pixel in a radar image, while a *ground pixel* is the corresponding surface area on the ground. Usually the dimension of a ground pixel is much larger than the wavelength of the radar. Based on the ratio of ground pixel dimension to the wavelength, the phase of an image pixel is generally the statistical sum of the phases of hundreds of elementary targets of the size of wavelength within the ground pixel. If the surface is uniform and homogeneous, these targets can be the same; otherwise are different. Interaction between the radar signal and the ground surface targets is a complicated process, involving scattering and absorption, depending on the dielectric constant and the target size. In equation (2), the complex wave vector is dependent on the dielectric constant, the slant range (distance between the antenna and the ground pixel). Thus, targets within the ground pixel of different dielectric constant will contribute differently to the phase of the image pixel [12]. Soil moisture and atmosphere will affect the dielectric constant and subsequently the wave number *k* during the wave propagation. The phase due to each target can rotate upon being scattered (depolarization) or be increased or decreased depending on the relative position of the target within the pixel [**R** in equation(2)].

### 1.4. InSAR and Interferogram

InSAR is a remote sensing technique using two or more SAR phase images acquired at different times to generate maps to detect and map changes of spatial and/or dielectric properties of the ground surface by using differences in the phase of the waves returning to the satellite or aircraft [1,3,4,6,13]. When a series of SAR phase images is available over a specific area, combining them into a series of differential interferograms allows us to follow displacement trends through time, resulting in multi-temporal InSAR. An InSAR *interferogram* is an image formed by the difference of two coregistered SAR phase images of the same area. A *fringe* is a line of equal phase in the interferogram. The number of fringes in an interferogram is counted from a reference point where the surface deformation (displacement) is supposedly zero. To count fringes in the regions where the fringes are extremely fine, the interferogram needs to be regenerated at higher spatial resolution by means of reducing the look number or using two images of shorter time interval. As discussed above, range can affect phase. Thus, an interferogram can be used to extract information such as landscape topography and its deformation patterns since the range changes affect the phase difference between two co-registered radar phase images.

The time interval between the two image acquisitions can vary from 0.1 s to years, depending on how fast the target is changing. The two phase images from which an interferogram is formed can be acquired either at a very short time interval (∼0.1 s) using single-pass interferometry or at two distinct times (up to years) using repeat-pass interferometry. The former case can be used for rapidly changing surfaces, and the later for slowly changing surfaces.

For the repeat-pass interferometry case, the two phase images must be properly coregistered. The requirement for the two coregistered images to form a meaningful interferograms is stringent, which means that the two SAR phase images for the same area must be precisely aligned (as accurate as 0.1 pixel) so that pixels in one phase image correspond exactly to the pixels in the other in geographic location. This is understandable because the phase difference between two different ground pixels does not make any sense.

The stability of the ground pixel, local slope of the terrain, the direction of observation, orbital configuration, frequency used for the two images, image processing procedure, topography difference observed from two slightly different points of view by the radar, all affect the formation of an interferogram. Thus, for two images to be used for an interferogram, the ground pixel should be stable, terrain slope should be small, and the observation direction, orbit configuration, and processing procedure should be exactly the same. For the stringent requirement for forming an interferogram, see [1].

### 1.5. Range Change Detection from Interferogram

If there is just a ground displacement of the whole pixel as a part of the image (without distortion of the pixel) along range direction - the *line of sight* (LOS) between the radar and the target, the displacement translates directly as a phase shift with respect to the rest of the image. Moving along range direction by half a wavelength for the pixel and thus a wavelength of round trip distance for the radar signal creates one fringe (2π phase difference). Therefore, one fringe in an interferogram corresponds to the displacement of half the wavelength in the ground displacement in the range direction. This is the principle how ground surface “deformation” is measured using InSAR [12].

As an interferogram is the phase difference image between two registered phase images, any factor that impacts the phase of a SAR signal will affect the interferogram. For instance, as the radar signal passes atmosphere, difference in the atmospheric status (atmospheric water vapor, clouds, etc.) between the two image acquisitions translates to different apparent path lengths. The effects due to the atmosphere are thus not canceled in differentiating the two phase images. Both ionospheric and tropospheric heterogeneities affect the interferometric phase.

For repeat-pass InSAR, the different acquisition geometry will result in different phase difference and thus affect the interferogram. For images acquired at different times on different orbits, the spatial separation of the two antenna positions is called the *baseline*. The orbital contribution to the phase difference is called “*orbital fringes*”. The ground surface topography also affects the interferogram. The topographic contribution to the phase difference is called “*topographic fringes*”.

Therefore, many factors - the distance between the antenna and ground pixel, surface topography, satellite orbits, the dielectric properties of the ground surface, atmosphere, and system noise [14], all affect the interferogram. For repeat-pass interferometry, the two registered images used to form the interferogram are taken from the satellite between subsequent passes with baseline B. The first image (reference image) is called *master image*, the second image is called *slave image*. The interferometric phase (phase difference between the two registered phase images) takes the form:

(3) $$\Delta \varphi =\frac{4\pi }{\lambda }\;\Delta D_{0}+\frac{4\pi }{\lambda }\;\frac{{\mathrm{HB}}_{⊥}}{R\;\text{sin}\;\theta }+\frac{4\pi }{\lambda }B_{//}+\Delta \varphi_{\mathrm{atm}}+\Delta \varphi_{\mathrm{dielectric}}+\Delta \varphi_{\mathrm{pn}}+2n\pi$$

where Δ*D*_0_ is the line-of-sight (LOS) displacement (surface displacement in the direction between the satellite and ground pixel), λ is the wavelength of the SAR system, *B*_⊥_ and *B_‖_* are the vertical and parallel components of the baseline orbit separation of the SAR image pair, respectively. H is the height of a pixel above a reference surface, R the slant range between the ground pixel and the antenna of the master image. *θ* is the local incidence angle, the angle between the radar beam and a line perpendicular to the surface at the point of incidence.

In equation (3), the first term represents the component of the interferometric phase due to the LOS range difference. As the two-component (amplitude and phase) SAR images are acquired from repeat observations over a certain time interval, this range difference can be due to the deformation of the surface during the time interval. Therefore, surface displacement can be measured from an interferogram if other terms in equation (3) can be determined.

The second term in equation (3) is the topographic effect. To calculate the topographic effect, an accurate digital elevation model (DEM) must be used. A synthesized interferogram is then generated from the DEM. To remove the topographic effect, this synthesized interferogram is then subtracted from the original raw differential interferogram. On the other hand, if other terms in equation (3) can be determined, a DEM can be derived from an interferogram.

The third term in equation (3) is the phase shift of the slave image relative to the master image because of a shift in the orbital trajectory between the two image acquisitions by *B_‖_* in the LOS. Phase shift due to parallel baseline *B_‖_* can be estimated by using precise orbits [15–16]. For European Space Agency (ESA) SAR data, the Precise Orbit Products provided by ESA is usually used as the initial parameters when calculating the orbital baseline. To remove the orbital effects, some researchers adjust the fringes in an interferogram to fit the displacements observed by GPS at seven geodetic bench marks [17].

Δ*φ_atm_* in equation (3) represents the atmospheric effect. Atmospheric effects should be corrected for the radar interferograms [6,14,18–21]; otherwise, radar interferograms will sometimes be misinterpreted. For instance, the large scale atmospheric delay observed at lower altitudes around a volcano could produce interferometric fringes in these areas that might be misinterpreted as indication of a large-scale inflation or deflation of the volcano. The atmospheric phase component is hard to calculate. A practical method to reduce the atmospheric effect in an interferogram as represented by equation (3) is to generate and combine multiple interferograms, a technique known as *stacking* [6]. Some permanent land markers such as rocky areas are very stable surfaces, the phase is preserved and thus the phase difference in an interferogram is always zero. This property of permanent scatterers can be made use of in removing atmospheric contribution to the overall phase difference.

Δ*φ_dielectric_* in equation (3) represents the effect due to changes in dielectric property of the ground pixel. For instance, dielectric constant changes due to soil moisture change. If other terms in equation (3) can be determined, soil moisture can be derived from an interferogram [22–23].

Δ*φ_pn_* in equation (3) is the phase noise. To reduce phase noise in an interferogram, adaptive filtering and multi-look techniques [1,24] are generally used or a weighted power spectral density filter and an adaptive filter are applied to each interferogram [24].

The last term in equation (3) represents the general 2π ambiguity associated with phase wrapping. Phase unwrapping is necessary to resolve this ambiguity. The aim of phase unwrapping is to find an estimate of the ‘true’ phase value given its principal wrapped value. A two-dimensional (2D) phase unwrapping is an important but complicated task. Even though it is impossible to completely unwrap the true phase, a wide range of methods have been developed to get good phase estimates [2,3,25–36]. Recent development shows that derivation of surface displacement can also be made without 2D phase unwrapping [37].

To derive the ground displacement from an interferogram, the effects due to stereo topography, base line, shift, atmosphere, dielectric property change of the ground surface, system noise, and phase wrapping can be corrected in principle according to equation (3). In reality, some residual phase error still exists. Orbital and topographic phase residuals are often removed by mainly adjusting the perpendicular baseline line component and yaw angle. If at least parts of the two images are coherent and the all other effects are removed, the remaining phase differences shown up as fringes in the final differential interferogram are the result of the *range* changes of displaced points on the ground [5]. Each fringe to the next one corresponds to a phase change of 2π. The total number of fringes is converted to displacement value (the projections along the SAR LOS of actual displacements) by multiplying by λ/2. This configuration makes InSAR capable of measuring ground-surface deformation with centimeter-to-subcentimeter precision at a spatial resolution of tens-of-meters over a large region. The LOS displacement Δ*D*_0_ is then converted to the horizontal displacement Δ*D* in the direction of displacement as:

(4) $$\Delta D={\Delta D}_{0}/\left(\text{sin}\;\theta \;\text{cos}\;\phi \right)$$

where *ϕ* is the angle between the direction of horizontal displacement and the projection of the radar looking direction on the ground range, as shown in Figure 1. Once the displacement is obtained, the average deformation rate is estimated by dividing the displacement by the time span between the two image acquisitions used to form the interferogram.

### 1.6. Selection of Interferometric Pair

The success of the technique of repeat pass interferometry depends largely on high correlation and coherence of the two sets of signals recorded during the two repeat passes if the scattering properties of the ground surface remain undisturbed between the repeats. Generally, selection of an interferometric pair is based on the sensitivity of the interferogram formed from the coregistered image pair to the topography as expressed by the altitude of ambiguity (*h_a_*) and coherence.

The *altitude of ambiguity* (*h_a_*) is related to the orbital separation between the image acquisitions, and equals the size of a DEM change that would produce one artifactual fringe. The *h*_a_ is given by [10,38]:

(5) $$h_{a}=\frac{\lambda R\;\text{sin}\;\theta }{2B_{⊥}}$$

Topographic effects can be ignored for image pairs whose *h*_a_ values are much higher than the estimated vertical accuracy of DEM.

*Interferometric correlation*, or *coherence*, that measures the variance of the interferometric phase estimate is usually calculated from the complex image pair. Coherence decreases with increasing system noise, volume scattering, and temporal changes, and therefore contains thematic information. Weathering, vegetation, random change in dielectric property within subpixel scale all affect the phase, leading to temporal decorrelation [39]. Coherence is thus a parameter that characterizes the quality of the interferogram formed from each pair of co-registered complex images. For each pair of such images, the correlation is estimated using the following equation:

(6) $$\rho =\frac{\left|\sum_{i=1}^{N}\sum_{j=1}^{M}C_{1}\left(i,j\right)C_{2}\left(i,j\right)*\right|}{\sqrt{\sum_{i=1}^{N}\sum_{j=1}^{M}C_{1}\left(i,j\right)C_{1}\left(i,j\right)*}\sqrt{\sum_{i=1}^{N}\sum_{j=1}^{M}C_{2}\left(i,j\right)C_{2}\left(i,j\right)}*}$$

where *C*_1_ and C_2_ represent the master and slave complex images, respectively. *C*(*i*,*j*) represents the complex value of pixel (*i*,*j*), where i represents the range direction and j represents the azimuth direction. *C*(*i*,*j*)* is the complex conjugate of *C*(*i*,*j*). N is the number of pixels in the range direction and M is the number of pixels in the azimuth direction, which are to be averaged to generate correlation of a single pixel that has different resolution from the original complex images. For instance, if the complex image has a range resolution of 20 m and an azimuth resolution of 5 m, setting N = 2, M = 4, equation (6) will generate a correlation image that has a pixel size of 40 m × 40 m, comparing to the original 5 m × 20 m pixel size.

### 1.7. Formation of an Interferogram

SAR data processing to form an interferogram includes: 1) conversion of the two raw data images to two single-look complex (SLC) SAR images; 2) coregistration of the two complex SLC SAR images to an accuracy of less than 0.1 pixel; 3) phase noise reduction - removal of radar system noise, effect due to image misregistration, and speckle effects caused by the complex nature of the imagery by applying a coherent average filter; removal of the effect due to local topographic slopes using pulse response filters; removal of atmospheric effect; and elimination of the topographic effect through subtracting the fringe pattern calculated from just an accurate DEM; 4) phase flattening; 5) interferogram reprojection; and 6) phase unwrapping. *Phase flattening* is a technique of removing the flat Earth phase function that can be calculated based on the image acquisition geometry from the actual phase function recorded in the interferogram. In areas where the elevation changes more rapidly (mountain regions), the frequency of the phase wrapping increases. Generally, the higher the wrapping frequency, the more difficult the area is to be unwrapped. Phase flattening can enhance phase unwrapping. However, in the areas where the wrapping frequency exceeds the spatial sampling frequency of the phase flattened interferogram, phase information is lost and phase unwrapping is not possible in the areas. Phase unwrapping is problematic due to fringe discontinuities caused by layover [40], areas of low coherence, and phase noise.

Two methods are usually used to form an interferogram: 1) The images are combined two by two using the digital elevation model elimination method. This method reveals fringes corresponding to contours of equal change in satellite-to-ground distance (i.e. range); 2) Method that uses three radar images [41] and does not require the use of an existing topographic mode [42]. For more detailed description of the procedure of interferogram, refer to the reviews [1,3].

### 1.8 Limits of Conventional InSAR and New Remedy

There are two conditions to be met in order to observe a fringe pattern. The first condition is that the spatial distribution and the electromagnetic properties of elemental scatterers contained within a pixel remain almost completely stable. A second condition is related to the difference between the two-way slant range distances, namely, measured along the radar LOS, corresponding to one pixel on the two SAR images in the interferometric pair. In particular, this condition requires that the difference between the two-way slant range distances between neighboring pixels has to be smaller than half of the radar wavelength λ in order to observe interferometric fringes without ambiguity [43].

The first limit of InSAR is related to temporal decorrelation [39], i.e. temporal stability of the spatial distribution of scatterers within a pixel. When the moisture and/or the freeze/thaw conditions of near surface layer change, or when surface is strongly disturbed, the spatial pattern of SAR sensitive scatterers within a pixel changes over time, resulting in random pattern of interferometric phase in a pixel neighborhood, or destruction of fringe pattern. Temporal decorrelation makes InSAR measurements unfeasible over such surfaces.

The second limit of InSAR is the spatial gradient or deformation time rate. If the relative displacement between two neighboring pixels exceeds one fringe, it cannot be detected using InSAR. Thus, the maximum detectable deformation gradient is one fringe per pixel. This is understandable since the phase difference value in a wrapped interferogram is between 0 and 2π. This means that the maximum difference in phase between two neighboring pixels is 2π, corresponding to one fringe (wavelength/2). For an interferogram formed from two images acquired at different times, this also translates to the maximum detectable deformation rate of one fringe per the time difference between the two acquisitions. For instance, if the second image is acquired N days later, then the average deformation rate should not exceed wavelength/(2N) days. If the average deformation rate exceeds this value, the two pixels from the two images are incoherent, no fringe will be generated. For a satellite, the *orbital cycle* is defined as the period of time when the identical orbit is repeated. If the orbital cycle for a specific satellite SAR system is M days, the maximum mean deformation rate by such a satellite’s SAR images is wavelength/(2M) days. For instance, for ERS-1/2 satellite of which its SAR wavelength is 5.67 cm, for its 35-day orbital cycle, the maximum detectable average deformation rate is 1.62 mm/day. If the tandem images of ERS-1 and ERS-2 are used to form tandem interferogram, the equivalent orbital cycle is one day, the maximum detectable average deformation rate can be increased to 56.7 mm/day. Phase unwrapping techniques can be applied and unwrapped interferograms, in which phase values vary from 0 to over hundreds of 2π, can resolve the limit of spatial gradient or deformation time rate.

An additional limit of InSAR is that the deformation amount inferred from the number of fringes are relative changes, not absolute changes relative to zero deformation. However, if a permanent marker can be identified in the image, this limit can be removed. To obtain absolute surface displacement, an assumed surface reference of zero (permanent marker) or known velocity needs to be identified in the interferogram. Since a fringe is a pattern, thus deformation can not be derived from a single pixel or a couple of pixels. Counting the number of fringes is much easier from an interferogram of larger area that not only include the deformed area where fringes are present, but also incoherent areas or permanent areas where phase values are always zero. This requires a large swath in both cross-track and along-track directions. If the spatial gradient is too small, that the whole swath is within one cycle, the interferogram cannot detect the change. Also for a specific image-acquisition system, sufficient magnitude and proper orientation of the deformation field is also imperative to be detected by the interferogram.

The multi-image Permanent (or Persistent) Scatterer (PS) technique deals with these problems in an innovative way [44–45]. The PS technique offers a systematic processing strategy, capable of utilizing all archived SAR data of a certain area from repeat orbits, and creating a stack of differential interferograms that have a common master image. Instead of analyzing the phase in a contiguous spatial domain, the phase of isolated points (permanent scatterers) with strong and stable radar returns is analyzed as a function of time, baseline, and space. The different spatio-temporal-baseline relations of the phase components of the PSs to topography, displacement, and atmosphere are used for successful separation of these different phase components in an estimation procedure. The invention of this technique was a big step forward towards a high accuracy observation of slow moving surfaces over long time spans, as it enables the identification, isolation, and estimation of millimeter surface deformation processes from space.

### 1.9. SAR Sensors for InSAR and InSAR Software

The first sensor in space for single-pass interferometry was the Shuttle Radar Topographic Mission (SRTM). Sensors for single-pass interferometry also include Topographic SAR (TOPSAR). Space-borne platforms from which SAR images can be used to form repeat pass interferograms include spaceborne imaging radar sensors decommissioned: Seasat (1978 - 1978) [46], European Remote-Sensing Satellite-1 (ERS-1) (1991–2000), Japanese Earth Resources Satellite (JERS-1) (1992 - 1998), Spaceborne Imaging Radar (SIR-C) (1994) and still in commission up to 2008: European Remote-Sensing Satelite-2 (ERS-2) (1995 –present); Canadian Radar Satellite RADARSAT-1 (1995 - present) [47], European Environmental Satellite (Envisat) Advanced Synthetic Aperture Radar (ASAR) (2002 - present) (http://envisat.esa.int/handbooks/), Japanese Advanced Land Observation Satellite (ALOS) phased-array type L-band (PALSAR) (2006- the present; http://www.palsar.ersdac.or.jp/), German TerraSAR-X (2007- the present; http://www.dlr.de/tsx/start_en.htm), RADARSAT-2 (2007 - present; http://www.radarsat2.info/), and Italian constellation COSMO-SkyMed (2007 - present) [46,48]. Because of the 1-day interval SAR acquisition capability from two identical C-band instruments onboard ERS-1 and ERS-2, the ERS-1/2 tandem mission [49] from 1995 to 2000 increased the probability of having a high coherence between the acquired data, making it possible for monitoring more rapid geophysical or biophysical processes. Additional spaceborne systems include the Spaceborne Imaging Radar-C/X-band Synthetic Radar (SIR-C/X-SAR) (operated for two 10-day periods during 1994). In addition, the 11-day NASA STS-99 mission in February of 2000 used a SAR antenna mounted on the space shuttle to gather data for the Shuttle Radar Topography Mission. Table 1 gives the specifications of these systems.

An interferogram can be computed with both free InSAR software packages and commercial packages. Free InSAR packages for academic uses include: 1) the Repeat Orbit Interferometry Package (ROI_PAC) developed at the Jet Propulsion Laboratory and the California Institute of Technology (JPL/Caltech), available at: http://www.openchannelfoundation.com/projects/ROI_PAC/index.html; 2) Doris (Delft object-oriented radar interferometric software developed by the Delft Institute of Earth Observation and Space Systems of Delft University of Technology downloadable from http://enterprise.lr.tudelft.nl/doris/; 3) Interferometric Processing System (IPS) developed by the Alaska Satellite Facility (ASF). Commercial packages include: 1) DIAPASON originally developed by the French Space Agency (CNES), now maintained by Altamira Information for both UNIX and Windows platforms (http://www.altamira-information.com/html/index.php); 2) GAMMA SAR developed by Gamma Remote Sensing for Solaris, Linux, OSX, and Windows platforms (http://www.gamma-rs.ch/software/) [50–51]; 3) IMAGINE InSAR embedded in ERDAS IMAGINE remote sensing software suite developed by Leica Geosystems Geospatial Imaging (http://gi.leica-geosystems.com/default.aspx); 4) Pulsar, developed by Phoenix Systems for UNIX based platforms (http://www.phoenixsystems.co.uk/); 5) SARscape, that was developed by sarmap s.a.; a Swiss company (http://www.sarmap.ch/). SARscape is interfaced with ENVI and can be run on Windows or Linux based personal computers.

## 2. Applications of InSAR

Repeat-pass interferometry allows the detection and mapping of the earth surface by using the temporal and spatial coherence characteristics, which can be successfully used for land cover classification, mapping of flooded areas, monitoring of geophysical parameters. The basic measurement of interferogram is the changes of spatial and/or dielectric properties using two images. The surface deformation and/or dielectric property change can be due to various forcings: earthquakes, landslides, lake or river surface water flow, oceanic water motion, movement of glaciers and ice sheet, accumulation of snow, forest canopy height, sand dune movement, dielectric constant change resulted from soil moisture change, freezing, or thawing, land subsidence due to ground water withdrawal, underground mining, hydrocarbon extraction, and permafrost melting, etc. The ground surface “deformation” can be anything the ground pixel is displaced between the two image acquisitions such as crustal deformation due to earthquakes, volcanoes, surface subsidence due to ground water exhaust or underground mining activities, river water level changes, etc. Therefore, applications of InSAR can be very broad – in seismology: land surface deformation due to earthquakes; in natural disaster monitoring and assessment: volcanoes and landslide movement; in surface water hydrology: water level monitoring, snow accumulation; in ground water hydrology: land subsidence due to excess water pumping or uplift due to ground water recharge; in glaciology: ice sheet motion and rheology, glacier flow, postglacial rebound of the lithosphere; in mining: land subsidence due to mining; in forestry: forest canopy height, forest mapping and monitoring; in environmental and structural engineering: ground subsidence and structural stability.

### 2.1. Application in Seismology

Coseismic deformation and postseismic relaxation of the lithosphere, if net surface deformations are present and fall within the limits discussed above between the two image acquisitions, should be detected by the interferogram. To detect the coseismic deformation due to an earthquake, the interferogram must be formed from two SAR images with one before and the other one after the earthquake. Since the technique can potentially measure centimeter-scale changes in deformation over time spans of days to years, it has applications for earthquake assessment and geophysical monitoring of earthquakes postseismic fault activity. Massonnet and Rabaute [52] formed the first earthquake interferogram from two ERS-1 SAR images taken before (April 24, 1992) and after (June 18, 1993) the Landers earthquake that occurred in California on June 28, 1992, illustrating the capability of InSAR in mapping the coseismic deformation field. Massonnet and Feigl [53] then formed an interferogram from two images acquired 40 days and 355 days after the main Landers earthquake and found a range shortening of 112 mm due to the Fawnskin earthquake, an aftershock in the Landers sequence that produced a coseismic bulge. The InSAR technique is now applied widely in characterizing the coseismic deformation field resulted from earthquakes, fault geometry and slip distribution [54–55], postseismic deformation and relaxation, and interseismic creep [38,56–60]. Cakir *et al.* [61] studied the February 24, 2004 Al Hoceima (Morocco) earthquake (*M_w_* = 6.4) using Envisat InSAR data and inferred that the 2004 earthquake took place most likely on a NW–SE trending right-lateral fault. Akoglu *et al.* [62] studied the May 26, 1994 (*M_w_* = 6.0) and February 24, 2004 earthquakes in the same region using ERS and Envisat InSAR data, respectively, and found that the 1994 earthquake being associated with N23° E trending left-lateral fault and the 2004 earthquake with N45°W trending right-lateral fault. Akoglu’s [62] results supported Cakir *et al.*’s [61] inference for the 2004 earthquake, which is in contrast with previous seismic interpretations. InSAR is now actively used by seismists to provide significant information on the main characteristics and location of earthquake ruptures, especially of earthquakes that took place on blind faults; and to estimate the optimal fault geometries and slip distribution [63–64]. The surface deformations derived from interferogram that indirectly reflect the brittle processes of an earthquake fault was used for dynamical fault model reconstruction [65]. A major limitation of InSAR for deformation studies is that the deformation is only one dimensional along the satellite’s line of sight, while most deformation is better characterized using three dimensional geodetic data. Many studies have incorporated multiple radar passes using different geometries to overcome this limitation (e.g. [66–68]). Recent developments of InSAR in deformation mapping include extraction of the 2-dimensional displacement using split-beam processing [69]. The availability of SAR data from new SAR systems such as ALOS PALSAR, Envisat ASAR, RARSAT-2, TerraSAR-X, and COSMO-SkyMed will enhance monitoring earthquakes and provide model constraints in seismological studies. Pritchard and Fielding [64] used the first InSAR data from the ALOS PALSAR data and the SAR data of wide swath mode of the Envisat ASAR to analyze a sequence of earthquakes on the subduction megathrust near Pisco, Peru. InSAR data analysis indicated that the main slip after the mainshock is about 70km from the pypocenter, suggesting a very low rupture velocity (< 1.5km/s) or a very long slip rise time.

### 2.2. Application of InSAR in Volcanology

Volcanic processes such as magma accumulation in subsurface reservoirs, magma transport and emplacement beneath volcanic structures will results in surface deformation, which InSAR can be used to detect. For instance, some offshore mud volcanoes in the South Caspian region possess shallow mud chambers which refill prior to eruption. The inflation of the chamber caused by the refill may produce measurable surface deformation that can be detected by interferogram [70]. Spatio-temporal evolution of volcanic processes can be derived from analysis of surface deformation derived from a temporal series of InSAR interferograms [71]. The measured deformation can then be used as constraints to inverse the depth, size, shape of the magma chamber (and pressure change) and magma supply dynamics, because point sources [72], dikes and sills [73–74] result in different surface deformation patterns in the modeling of deformation caused by magma intrusion [75–78]. Therefore, interferogram-derived deformation is widely used as constraints for modeling studies of volcano deformation to determine magma sources and dynamics [71,79–80], to identify faults caused by the volcanic activities [81–82].

Knowledge of temporal and spatial distribution of magma accumulation, transport, and emplacement can help locate magma reservoirs and the formation of eruptive vents, pave the path of understanding volcanic processes, development of sophisticated and comprehensive models for magma intrusion, recharging, and volcano spreading, and competent forecasting of future volcanic eruptions, flank instabilities and sector collapses. GPS is one of the most suitable techniques to measure ground surface deformations because of high accuracy and provision of three dimensional components of deformation field. But the limitation of GPS station density (GPS stations per unit area) and the continuous coverage of interferogram make the integration of InSAR and GPS a useful approach to map highly accurate deformations (i.e. at sub-centimeter levels) with unprecedented spatial coverage. Interferogram alone, or in combination with other data sets of such as GPS, geodimeter, and micro-gravity data are now widely used for pre-, during-, and post-eruption observation of volcanoes. Examples include the measurement of deflation induced by the activation of Etna volcano from May 17, 1992 to October 24, 1993 by Massonnet *et al.* [83], the measurement of the deformation field associated to the 1997 eruption of Okmok volcano in Alaska [84] and the Campi Flegrei caldera activation [85], observation of volcanic uplift and ‘trapdoor’ faulting [81], monitoring of deep accumulation of magma and large-scale inflation in the early development of magmatic systems before eruption [80,86–89], caldera deflation and volcano contraction [90], regional subsidence due to volcanic eruption [81,91], magma intrusions and edifice radial spreading after eruption during magma-recharging phase [92–93], subsidence of dormant volcanoes [94], and magma chamber geometry estimation [95].

Fialko *et al.* [96] used the interferogram data to constrain the dynamics and morphology of the deformation source responsible for the 1997–1998 inflation crisis in the Long Valley caldera (California). Joint inversion of the interferogram and two-color geodimeter data for the period between 1996 and 1998 suggests that the deformation source has the shape of a steeply dipping prolate spheroid with a depth of 7 km, major and minor axes of 4.2 and 1.8 km, respectively, and an excess pressure of several megapascals. Using InSAR, deformation field of Nisyros volcano (Greece) was studied by several authors [97–100]. The seismic unrest without eruption started at the end of 1995, peaked in August 1997, and ended at the end of 1998. For these studies, InSAR provides the only tool to assess the deformation fields of the volcano before 1997 when the first GPS stations were set up. Sykioti *et al.* [100] showed that a ground uplift of 84mm in the slant range direction was observed between June 1995 and May 1996 and further uplift of 56mm from May 1996 to June 1997. A subsidence of 42 mm was observed between June 1997 and June 1999. The change from inflation to deflation most likely took place at mid-1998. Lagios *et al.* [97] further study showed that a deformation of at least 56mm along the slant range appeared for the period 1996 through 1999, which is consistent with modeling results using a two-source model constructed to fit the GPS observed deformation. Ruch *et al.*’s [89] study on the Lazufre volcanic region (Chile), where no deformation was detected previously, shows that an uplift of the region of up to ∼3 cm·yr^−1^ during 2003–2006 was observed on a large scale (1100 km^2^) using interferogram data.

Amelung *et al.* [81] constructed temporal deformation maps of volcanoes with or without eruption in the Galápagos Islands using satellite radar interferometry. Uplift rate for each volcano, which is a good indicator of magma accumulation, is then derived from these maps. These maps help in identifying inflation, co-eruptive deflation and shallow dyke intrusion of volcanoes with eruption; and in identifying inflating sill and ‘trapdoor’ faulting of volcanoes without eruption. Using GPS and interferogram data, Geist *et al.* [90] shows that during the 8 days of the 2005 eruption of Sierra Negra volcano, Galápagos, Ecuador, the caldera floor deflated by about 5 m, and the volcano contracted horizontally by about 6 m. Based on these estimates, the total eruptive volume is estimated to be about 1.50×10^8^ m^3^. Abidin *et al.* [91] used InSAR, in combination with GPS, studied the subsidence and uplift of a populated area of Sidoarjo, East Java are due to the eruption of the Lusi mud volcano during 2006–2008. They found that the earth’s surface has been subsiding at rates of 0.1–4 cm/day. Maximum rates of subsidence occurred in an area 300–400 m to the northwest of the main mud volcano vent. Horizontal displacements were 0.03–0.9 cm/day and were also towards this area. In general uplifts of up to 0.09 cm/day were recorded in areas outside of the edifice. Changes in elevation measured using interferogram provide regional datasets of subsidence/uplift. de Zeeuw-van Dalfsen *et al.* [101] using interferograms combined with micro-gravity and GPS data as constraints to generate a model for the processes operating at Krafla volcano and show that the rate of deflation at Krafla is decaying exponentially, suggesting a drainage rate of ∼0.23 m^3^/s.

Lundgren *et al.* [92] studied the post-eruption deformation evolution of Mount Etna volcano from 1992 to 2001 by more than 400 temporal radar interferograms from ERS-1/2 data formed using the small baselines subset (SBAS) technique [102]. Their results show that during this time interval Mont Etna volcano experienced magmatic inflation and radial spreading to the West, South, and East, suggesting gravitational spreading of the volcanic edifice; and growth of a southeastern basal anticline, suggesting deep-seated magma intrusions and edifice spreading. Palano *et al.* [93] combined InSAR and GPS data to study the ground deformation field at Mount Etna volcano during the magma-recharging phase over 1993–2000 after the 1991–93 eruption. Their results show inflation since 1993, a deep intrusion on the western flank between March and May 1997, a general deflation at the upper part of the Volcano from 1998 to 2000, and a continuous eastward to south-eastward motion of the eastern sector of the volcano. Previous studies of the deformation fields of Mont Etna volcano include [83,103–107]. These studies will help understand the volcanic processes, especially the synergic action of various forcings such as gravity, magma forcing, dyke intrusion, and regional tectonics in the evolution of an active volcano.

### 2.3. Applications of InSAR in Land Subsidence and Landslides

#### 2.3.1. Land Subsidence and Uplift

Land subsidence, lowering of the land surface by a variety of subsurface displacement processes, affects an aggregate area of more than 44,000 km^2^ in 45 states in the U.S. [108]. Common causes of land subsidence include sediment consolidation due to its own weight and tectonic movements (geological or natural subsidence), withdrawal of ground water and geothermal fluid, oil and gas extraction from underground reservoirs; dissolution of limestone aquifers (sinkholes); collapse of underground mines; drainage of organic soils; and initial wetting of dry soils (hydrocompaction). Measuring the spatial and temporal changes in the subsidence pattern using interferogram can provide constraints on the permeability and compressibility properties of the compacting formations, which are important parameters for predicting future subsidence.

Land subsidence can be caused by groundwater pumping, which results in compaction of aquitards during the slow process of aquitards drainage. Subsidence detected from interferogram can be used for parameter estimates in simulations of aquifer system compaction [109]. Subsidence from excess ground water withdrawal has impacted costly on both the operation and design of canals in California [110]. The spatial scale of the land subsidence can be well monitored using InSAR. For instance, during 1950–1970, the city of Venice (Italy) was affected by serious land subsidence with highest rates due to groundwater withdrawals [111–113]. Since 1970, drastic measures to reduce both industrial and other artesian extractions were taken to stop the subsidence due to ground water withdrawal. But subsidence due to consolidation processes may still be continuing. Carbognina *et al.* [111] showed that the vertical displacement rates are between + 1.0 and −2.0 mm/year as derived from interferogram images ERS-1/2 from 1992 to 1996. This indicates the quasi-stability of the sediments in the landward and central part of the city during the time period of time, but some small zones subsiding at a rate greater than 1 mm/year in western and eastern areas consolidation processes may still be continuing. This type of subsidence due to groundwater pumping was also observed in other cities in Italy [114]. In semi-arid and arid regions, ground water is the main water resources for civilian use and agricultural irrigation. Excessive withdrawal of ground water can cause large scale subsidence. InSAR application will continue to be very useful in monitoring the land subsidence due to excessive withdrawal in the next decades. Conversely, during a wet season, ground water recharge makes the ground-water levels to rise. Originally unsaturated sediment shifts from the granular skeleton to the pressurized pore fluid, causing the skeleton to expand and the surface to up lift. From interferogram analysis, Hoffmann *et al.* [115] showed seasonal subsidence and uplift in Las Vegas valley, Nevada. Lu and Danskin [116] successfully detected an uplift of several centimeters in the Bernardino basin of Southern California due to ground-water recharge using interferogram analysis of ERS-1 and ERS-2 images. These seasonal deformation data contain important information about the hydrogeologic properties of the aquifer system and are of considerable value in assessing the effectiveness of ground water recharge programs. Using InSAR, Bawden *et al.* [117] showed that seasonal fluctuations of 50 mm of basin uplift due to ground water recharge during the wet winter season, and 60 mm of subsidence due to ground water withdrawal during the dry summer season in Santa Ana basin, California, with the greatest fluctuations near the city of Santa Ana and at the northwestern end of the basin. Similar analysis for the Los Angeles basin by Watson *et al.* [118] verified that vertical motion related to annual variations in the elevation of the water table needs to be taken into account when interpreting the geodetic data for tectonic motion. Schmidt and Bürgmann [119] used 115 differential interferograms over the period from 1992 to 2000 to study the spatial and temporal pattern of uplift of the Santa Clara Valley aquifer so that both seasonal uplift and subsidence and long-term uplift can be resolved. The seasonal surface deformation observed reflects the poroelastic response of the confined aquifer resulting from the redistribution of groundwater, while the long term uplift reflects the net increase in pore fluid pressure. Results from Schmidt and Bürgmann’s [119] study showed that the recovery of groundwater levels in the Santa Clara Valley which began in the 1960s appears to have continued through the 1990s as inferred from the net regional uplift. The knowledge of spatial and temporal pattern of deformation of an aquifer will help understand the mobility of groundwater within the aquifer, the distribution of permeable units, and the mechanics of the aquifer system.

Warm or hot ground water in the areas of hot springs or volcanic zones is a useful energy resource. Hot water is pumped for its geothermal energy. Like ground water exhaust, however, excess pumping of the ground hot water at a speed higher than the recharging rate will cause ground subsidence both vertically and horizontally, in severe cases can damage property and environment [120–121]. Land subsidence is induced by volume changes or reduction of subsurface pore pressure in the geothermal reservoir by depletion of fluid storage as well as thermal contraction. Monitoring the subsidence in the geothermal field using precise level network or interferogram is important so that measure can be taken to prevent damage to infrastructure and environment. Massonnet *et al.* [122] formed an interferogram from ERS-1 radar images spanning a period of two years near the East Mesa geothermal plant in southern California and found as much as 90 mm of subsidence. This subsidence is due to the withdrawal of geothermal fluid produced from medium to fine grained quartzose sandstone. The total volume loss due to subsidence was estimated to be 3.6×10^6^ m^3^, in good agreement with a total fluid removal of about 5.0×10^6^ m^3^ from the geothermal reservoir. The same technique is used to monitor the deformation of other geothermal fields in Iceland [123]; in Cerro Prieto, Mexico [124]; in Coso, Calfornia [125–126]; and in Taupo Volcanic Zone of New Zealand [120]. The objective of mapping the subsidence field of the geothermal fields using InSAR is four folded: 1) to assess the environmental and structural damage due to excess extraction of geothermal fluids; 2) to derive important parameters such as permeability and compressibility properties of the compacting formations, the scale and rate of subsidence so that the future subsidence rate and scale can be predicted; 3) to identify areas of infrastructure potentially at risk of structural damage and to reduce the impact of future development of the geothermal fields; and 4) to develop future strategy including control of extraction rate and reinjection of fluids back to the geothermal fields to minimize environmental impact. The environmental impact of subsidence due to excess pumping can be substantial. For the East Mesa geothermal field, there are two canals at the vicinity of the subsidence field; further subsidence will disturb the normal operation of these canals. Monitoring of the subsidence is usually accomplished using leveling network. However, the scope of the leveling benchmarks is often limited. InSAR measurements cover area far beyond the boundaries of the geothermal systems and beyond the scope of the leveling benchmarks. Thus, InSAR makes it possible to interpret the subsidence signals in the context of a wider regional deformation and to identify areas of infrastructure potentially at risk of structural damage and to reduce the impact of future development of the geothermal fields within a much larger area than ground leveling network can cover [120].

Subsidence of land surface can also be caused by mining activity. Land subsidence due to mining is ubiquitous. Underground coal mining has occurred beneath 8 million acres of land in the U.S., more than one quarter of which experienced subsidence and thousands of acres in urban areas are threatened by subsidence; subsidence from other metal and nonmetal underground mines affected 17,000 acres [127]. Subsidence above a mine results from readjustment of the overburden and is thus time-dependent. Some movements take place during mining and some as early as a decay after mining and as late as centuries, depending on the mining method (room and pillar mining, longwall mining, etc.), thickness of coal mined, mining geometry, the thickness and geological characteristics of the overburden and the mine floor, etc. [127–128]. Monitoring of rate and extent of subsidence above a mine is to provide important information on surface developments designed to minimize the impact and to make sure if subsidence is complete before construction of surface structure can be carried out. Similarly, the amount of subsidence can be derived from the number of fringes on an InSAR interferogram. For instance, noticeable subsidence around the underground coal mining near Gardanne in southeast France has occurred since the 1960s, when intense coal mining due to mechanical extraction began. Waste rock is used to refill the cavities behind the extraction front to reduce collapse hazard, but cannot eliminate completely the subsidence. Using InSAR interferogram formed from ERS-1 images, Carnec *et al.* [129] showed that in the underground coal mining field near Gardanne, France as much as 42 mm subsidence could occur in 35 days. Further interferometric monitoring of subsidence in the coal mining field from images acquired by both European ERS-1/ERS-2 satellites between 1992 and 1995 revealed the migration of the subsidence halo caused by the advance of the coal working face [130]. Since the underground cavity due to such underground mining activities can be triggered by local seismicity, environmental impact due to the potential hazards can be more conveniently monitored by precisely mapping the spatial extent of the subsidence. Deformation was also recorded by two separate leveling lines covering the same period of time. The rms scatter in vertical displacement between the radar measurements and the two leveling lines is 10 mm and 16 mm. The interferometric study adds to existing surveys. These demonstrations open wide prospects for industrial and environmental applications with both economic and legal consequences.

Land subsidence can also caused by gas/oil extraction. Using InSAR, Bawden *et al.* [117] found that the Wilmington and the Salt Lake oil fields (California) underwent an episodic subsidence rate of 28 mm·yr^−1^ and 11 mm·yr^−1^, respectively, during 1997 to 1999; while the Santa Fe Springs and portions of the Baldwin Hills (BH) oil fields uplifted at a rate of 5–9 mm·yr^−1^. The uplift of the Santa Fe Springs oil field was due to fluid injection, though the uplift mechanism for the Santa Fe Springs oil field is unclear. Using interferogram over the oil field of Bakersfield, California, Snieder *et al.* [131] showed that as much as 3 cm land subsidence was observed in the northwest region and the southeastern region over an area approximately 5 km wide with as much as 5 cm of subsidence in two localized features on the western margin of the subsidence trough. All the subsidence observed in these examples was caused by oil extraction. Stancliffe and van der Kooij [132] used the InSAR technique to monitor production activity at the Cold Lake heavy oil field (Alberta, Canada). They generated contour maps of elevation change from JERS-1 interferograms over the oil field at 43- and 86-day intervals. At an area where steam was being injected into the reservoir to mobilize the bitumen, up to 0.19 m in elevation gain during 43 days was derived, while at an area where the steaming has been completed and the pads brought onto production, subsidence on the order of 0.17 m was estimated. At the Cold Lake oil field, the injection of steam causes the pump jacks to heave and subside by as much as 30 cm during the first steam cycle. Such studies on the correlation between production activity and surface deformation may help understand the oil production and at the mean time reduce the environmental impact.

#### 2.3.2. Landslides

InSAR appears attractive for landslide hazard investigations and possibly for preliminary warning. However, the steep and rough topography typical of landslide-prone areas, relative large rates of movement causing phase ambiguity problems and signal decorrelation, together with the fact that local atmospheric variations can be particularly pronounced in regions with strong topographic relief, leading to strong atmospheric phase artifacts, often hamper the interferometric pre-processing, making it difficult to estimate displacements [1,6,43,133]. Thus, in many cases, the spaceborne interferometric monitoring of mass movements was suggested to be used together with ground-based SAR interferometer systems [134]. In order to overcome the problems associated with decorrelation noise caused by random temporal variations of terrain reflectivity and atmospheric delay, one possibility is to apply the technique of “permanent scatterers (PS)” [44–45] that allows to obtain millimeter-level accuracy displacement measurements over isolated stable points in the scene. Successful applications of InSAR technique to landslide monitoring and slope instability studies include [45,133,135–142]. Catani *et al.* [43] demonstrated that on average, the slope angles computed via interferometric methods are generally in better agreement with landslide locations than DEM slope angles, indicating the usefulness of InSAR to help categorize the landslide hazard levels of slope.

### 2.4. Applications of InSAR in Glaciology

Glaciers and ice sheets are sensitive to internal instabilities or climate fluctuations. Continuous retreat of glaciers and disintegration of ice shelves cause concerns of rise of sea level and severe weather due to changes of climate patterns [143]. To understand well the dynamics of glaciers and physical mechanism of motion of ice streams and ice sheets, accurate measurements of displacement and subsequent velocity field of glaciers and ice shelves are important because they provide a better knowledge of the rheological parameters that control the flow of glaciers and ice shelf. For instance, using an inverse control method, Larour *et al.* [144] inferred the ice rigidity of the Ronne Ice Shelf that best matches the ice velocity field derived from interferogram. Through measurement of displacement and velocity field of glaciers, InSAR has been used successfully to monitor glacier surges [145–146], ice sheet motion [42,147–149], ground lines of marine ice sheets [42,150], ice sheet topography [148,151], glacier ice-flow velocity field [3,147,152–156] and motion patterns [37], and flux [153,155–157], patterns of wind-drifted snow [158] and snow accumulation [159], uplifting [160] and infilling [161] of ice cauldrons, the effects of the subglacial floodwater on glacier flow during draining episodes [162], and calibration of numerical modeling of ice shelf flow and dynamics [163]. Vertical displacement features derived from interferogram have been used to infer subglacial water movement [145,164]. InSAR has also been used to infer the water level or volume change of supraglacial lakes through DEM construction and then comparison of height change [165–166]. Combining two SAR phase images with 6-day separation, Goldstein *et al.* [42] used InSAR to measure the flow velocity field of the Rutford Ice Stream, Antarctica. In the following years, InSAR has been extensively used for mapping glacier surface velocity (e.g. [1,167]). Joughin *et al.* [153] and Mohr *et al.* [155] combined ascending and descending passes of the satellite and added constraints on the ice flow to obtain all three components of the displacement vector. Combining cross-correlation techniques with InSAR, 2D ice-flow velocity field can be obtained [152,168]. Glacier velocity data derived from interferogram are used to assist locating the supraglacial lakes for glacial hazard management [169]. Glacier velocity field derived from interferogram can also help identify the causes that result in ice shelf acceleration [163].

For monitoring of mountain glaciers [37,170], icefalls or marginal shear zones of glaciers, crop growth [171], short periods of time (1–3 days) of successive SAR images are required. However, no present or planned satellite missions have such short repeat acquisition plan. Thus, it would be difficult to measure the motion of mountain glaciers, ice falls, using InSAR. To overcome this difficulty, optical images can provide an alternative to interferogram [170] for the measurement of ice flow of glaciers. Products derived directly from interferogram will be combined with other science products, for instance, DEM from altimeter for mechanistic studies. One example is the combination of ice flow velocity derived from interferogram in combination of altimetry data to determine the mechanism of the inland thing of Pine Island Glacier, West Antarctica [172]. It is expected more discoveries from such combination will occur in the future.

Non-polar and alpine-style glaciers are recognized as being particularly sensitive to the current trend of climatic warming. Mountain glaciers are usually associated with small sizes but larger deformation rates than large ice caps, ice fields, and the Greenland and Antarctic ice sheets. Due to the displacement gradient (i.e. strain) threshold of InSAR, only a limited number of studies [156,168,173] have successfully measured the ice-flow velocity field with InSAR. For this reason, monitoring icefalls or marginal shear zones of glaciers and mountain glaciers using InSAR requires short periods of SAR images acquisitions so that correlation between the two images are preserved. The ERS-1 ice phase (3-day orbital cycle) and the ERS-1 and ERS-2 Tandem Mission (1 day separating the passes of the satellites) can serve the mission well to derive velocity fields of glaciers. InSAR DEMs are also used to monitor thinning of glaciers [174].

The identification of patterns of glaciers may be improved in the area by the Differential Interferometric SAR (DInSAR) technique [41,58]. It uses the interference of electromagnetic waves to measure precise distances or deformation [175]. However, more ERS-1, ERS-2, and Radarsat SAR images are needed to generate more interferograms to meet this research goal. When two coherent single-look complex SAR images acquired by repeat satellite passes on different dates are co-registered precisely and the difference of the phase values of individual pixels on the two SAR images are calculated pixel by pixel, an interferogram is formed. The resulting interferometric phase of each pixel includes components of topography and surface deformation. The topographic component of the interferometric phase can be removed using an existing DEM [58] or other interferograms [41] through DInSAR techniques. When the separation of the satellite orbits (orbital baseline) is small, the topographic component of the interferometric phase is not sensitive to errors in DEM. Using DInSAR techniques, accurate motion patterns of glaciers elsewhere have been derived [145,176]. The ASF software package - InSAR Processing System (IPS) which was designed for generation of digital elevation model (DEM) has been slightly modified to become a DInSAR tool, which has been used successfully in a preliminary study of the glaciers in this area.

### 2.5. Application of InSAR in Hydrology

#### 2.5.1. Soil Moisture Monitoring

Estimates of erosion, deposition, soil moisture, water level change, and net volumetric change of discharges may be achieved by using InSAR [131,166,177]. The dependence of the backscattered signal intensity to the soil moisture is widely documented in the literature. However, very little has been done concerning the sensitivity of the signal phase to the soil moisture conditions. Even if it was not possible to determine a simple relationship between the phase and the soil moisture profile (as the relationship varies according to the frequency, the roughness, the moisture level and the shape of the moisture profile), general trend of the soil moisture influence on the correlation coefficient was put on the forefront. Soil dielectric constant not only affects the amplitude of radar backscattering but also the phase of backscattered radar echoes. Since the dielectric constant of water is much higher than that of the dry soil matrix, soil moisture difference in the ground when the two radar images used to form an interferogram are acquired results in difference in phase due to the difference in dielectric constant. With the aid of Radarsat-1 images and genetic programming model, Makkeasorn *et al.* [178] showed the average volumetric soil moisture is 15.5 % in the September 2004 in Choke Canyon Reservoir watershed, Texas. It may be extended to monitor the soil moisture difference in the future using InSAR.

Gabriel *et al.* [12] formed a color-coded interferogram of agricultural fields in the Imperial Valley, California from three radar images of L band (25 cm) acquired by Seasat on three separate dates spanning 12 days in 1978. The interferogram indicates phase changes associated with watering. The phase changed by up to 0.3 cycles on agricultural fields watered by irrigation canals in the time between the radar images [12]. The phase change translates to an equivalent range change of 3.75 cm. The phase change is due to the dielectric constant difference caused by water content in the soil. An equivalent range change of 1 cm can be derived from the interferogram. Short-term interferogram on agricultural fields due to soil moisture was also observed in the Ukraine. Nolan *et al.* [22–23] used ERS-2 interferogram data in a high-plains region of Colorado and showed that the observed millimeter-order of change in path length may be caused by variations in soil moisture of a few percentages in volume. The mechanism of the water content in soil on the radar phase change is still controversial [1,12,22–23], but the equivalent range change is related to the amount of water in content will provide a potential useful tool for soil moisture monitoring.

#### 2.5.2. Water Level Measurement and Monitoring

In principle, temporal river discharge and the water level variations in lakes or reservoirs can be calculated from purely geometric measurements of river cross sections, water levels, and surface slopes for both rivers and lakes or reservoirs [179]. Thus, water level (stage) for lakes/reservoirs and major rivers can be retrieved from spaceborne remote sensing instruments that have the capabilities of geometric measurements [180]. These instruments include imaging radars. Spaceborne water level measurement is greatly improved with current measurement [180–182], which was in turn demonstrated by using along-track InSAR (ATI) techniques [182–183]. For example, if there are persistently existing reflectors (e.g. leafless trees) sticking out of a water surface, interferograms can be formed even over the water that can result in cm-scale estimates of changes in water level [183–186].

### 2.6. Applications in Forestry

In forestry, canopy height is often used to estimate forest biomass and above-ground forest carbon stock as the quantities are allometrically related. The phase of a specific pixel containing vegetation depends on vegetation structure, scattering mechanisms, and sensor characteristics (wavelength, polarization, looking angle, etc.). Because the phase of a pixel is the sum of the returns from a collection of scatterers including stems, branches, twigs, leaves or needles, trunks, and surface soil, the types of scatterers interact most strongly with the radar wave depend on the wavelength, polarization, incidence angle, and vegetation subpixel fraction. Forest canopy height can be estimated using InSAR, including polarimetric interferometry (PolInSAR) [187], because the interferometric phase relates to terrain height and vegetation canopy height [2,188–190]. Balzter [2] provides an overview of the potential and limits of InSAR for applications to forest mapping and monitoring. Recently, polarimetric SAR interferometry (PolInSAR) has received increasing interest for forest monitoring and mapping [187–189]. To accurately map the canopy height, phase unwrapping is always important [25,26,28,34]. The use of InSAR for estimating canopy height was demonstrated using C-band interferometric height discontinuities at forest edges by Hagberg *et al.* [191] and using effective interferometric C-band height from ERS-1 by Askne *et al.* [192]. Rignot [193] studied tropical rain forest using dual-frequency interferometric SIR-C shuttle radar data at C- and L-band. Andersen *et al.* [194] reported that the airborne dual-frequency SAR system TopoSAR consisting of a single-pass X-band and a repeat-pass P-band interferometric SAR constellation can be used to estimate mean stand canopy height, canopy fuel weight and other biophysical parameters. Dual-frequency InSAR method involves the generation of a digital terrain model (DTM) underneath the forest canopy from a repeat-pass long wavelength (e.g.; L-band, P-band). InSAR data taking advantage of its capability of penetration through canopy [195]. The DTM is then subtracted from the single-pass short wavelength band (e.g., X-band) interferometric surface height to obtain an estimate of the forest canopy height with appropriate adjustment for penetration depth that is vegetation type dependent.

The availability of an accurate terrain model is often a limitation of this method. Balzter *et al.* [188] estimated the forest canopy height based on a canopy height model derived from dual-wavelength InSAR with airborne X-band VV polarized single-pass and L-band HH polarized repeat-pass SAR interferometry using data acquired by the E-SAR sensor over Monks Wood National Nature Reserve, UK. Then, the forest carbon pools were estimated following allometric methods applied to the remotely sensed canopy height models with appropriate allometric adjustment [196–197]. When compared to the canopy height measured from LIDAR, the relative error for the canopy height derived from dual-frequency InSAR is 28.5%. The rmse of carbon content per hectare contain error components from the canopy height estimation but cannot be fully quantified [188]. The dual-wavelength InSAR technique could be applied to data from a constellation like the TerraSAR-L and Tandem-X missions.

Besides the capability of estimating canopy height and subsequent carbon content, InSAR can also be used to improve biomass estimation from radar backscattering coefficient because the fringe frequencies of the interferogram can be used to correct radar backscattering coefficient for terrain effects and thus improve radiometric calibration [198]. Other applications of InSAR in forestry include classification of forest types and land cover [19,192–193,199–202].

## 3. Discussion and Outlook

InSAR is a relatively new remote sensing technology. Through such a thorough literature review, the breadth and depth of this technology become lucid for users. There are several ways to further promote the potential of InSAR and possible establishment of InSAR satellites designed for operational InSAR application missions [203]. From a technological point of view, removal of the atmospheric effect is always a hard task in applications of InSAR in deformation detection. The persistent scatterers (PS) technique [44–45,204] seems very promising in removing atmospheric effect without knowing the atmospheric status [137,205]. This technique can also enable removal of DEM and orbital errors from each interferogram. These methods rely on the existence of persistent scatters - objects that remain highly coherent through time. However, the scarcity of natural persistent scatters makes areas containing many potential coherent targets such as buildings as the most favorable region for deformation detection. Thus, an establishment of a world-wide network of PS in the future will obviously benefit the deformation mapping worldwide.

Detection of rapid surface displacement using InSAR is often inhibited from the long repeat cycle. Except for bare and sparsely vegetated fields, the interferometric correlation decreased too much after a 35-day period of ERS-1 or ERS-2. The tandem mission of ERS-1 and ERS-2 [206] and shuttle-based SRTM mission demonstrated the necessity of contemporaneous acquisition of image pairs for quality interferogram formation by reduction of temporal decorrelation. Because of the ERS-1 payload switch off and the phase shift between ERS-2 and ENVISAT SAR sensors, the InSAR tandem acquisition is compromised for the near future. In this aspect, synergies among different satellite images should be enhanced in the future. Very positive results from ERS-1’s operation at three-day repeat cycle in 1994 and the tandem mission of ERS-1 and ERS-2 in 1995–1996 demonstrated that in the future, such missions of short repeat cycle is not only necessary, but imperative for the success in monitoring surface changes at rapid speed at centimeter or millimeter accuracy.

As already demonstrated for the monitoring of land subsidence [207], landslides [142], surface deformation in heavily vegetated and seasonal snow covered terrain [19,208], and active rock glaciers [209–210], L-band SAR has the capability of complementing the existing applications based on C-band data. In the case of rapid displacements, the larger wavelength reduces signal decorrelation and phase unwrapping problems. Furthermore, the greater penetration of the radar signals into the snow and forest at L-band compared to C-band [211] results in a reduced temporal decorrelation. It is thus expected that L-band PALSAR data from the ALOS and TerraSAR-L missions would contribute to this type of research.

More technical developments and applications of InSAR are expected with the data available from fully-polarized SAR sensors (ALOS, Radarsat-2, TerraSAR-X, TerraSAR-L, etc). Polarimetric SAR interferometry will enhance mapping of land cover and forestry [187–189,212]. With more InSAR data with short repeat cycle in place, more applications of InSAR are expected in seismology, volcanology, glaciology, hydrology, natural hazard monitoring and assessment, land subsidence due to thermal fluid pumping or ground water withdrawal or mining, oceanography, forestry. We expect the applications of InSAR will expand in other disciplines include archaeology and cultural heritage preservation [213], mountain uplift monitoring that is related with tectonics [214], production monitoring of oil fields [132], monitoring of injection site of geological CO_2_ sequestration [215] in the near future. Although InSAR is very useful for monitoring the surface deformation, operational use of InSAR for such activities is still years away [203]. Limited SAR data suitable for interferometry often makes a thorough data analysis for a specific application difficult [70]. The many discoveries from the limited number of past SAR sensors are just eye-opening. Dedicated satellite missions with short repeat cycles are imperative for integrated studies and operational monitoring of natural hazards (earthquake, volcano, and landslides), glacier and polar ice sheets, and large-scale forestry resulting from climate change. Cyranoski [216] and Donnellan *et al.* [217] reported the earth scientists’ aspiration of such missions, which becomes even stronger.

## Figures and Tables

**Table 1.** InSAR systems and characteristics.

**Sensor**	**Repeat cycle (days)**	**Wavelength (cm)**	**Resolution (m) (azimuth × ground range)**		**Look angle**
ALOS/PALSAR	46	23.62 cm	Fine mode 1	**10m × (7–44m)	8°–60°
			Fine mode 2	**10m × (14–88m)	
			*PL mode	**10m × (24–89)m	8°–30°
			*SC mode	100m × 100m	18°–43°
COSMO/SkyMed	16	3.125cm	*SL mode	<1m	>25°–50°
			*SM mode	<3–15m	>25°–50°
			*SC mode	< 30–100m	>25°–50°
Envisat/ASAR	35	5.63 cm	Image mode	^#^30m × 30m	15°–45°
			*AP mode	^#^30m × 30m	15°–45°
			*WS mode	150m × 150m	17°–42°
			Wave mode	10m × 10m	15°–45°
			*GM mode	1km × 1km	17°–42°
ERS-1	3, 35, 168	5.66 cm	30m × 30m		20°–26°
ERS-2	35	5.66 cm	30m × 30m		20°–26°
JERS-1	44	33.53cm	18m × 18m		35°
RADARSAT-1	24	5.66 cm	Fine mode	9m × (8,9)m	37°–47°
			Standard mode	28m × (21–27)m	20°–49°
			Wide mode	28m × (23,27,35)m	20°–45°
			*SC narrow mode	50m × 50m	20°–49°
			*SC wide mode	100m × 100m	20°–49°
			Extended H mode	28m × 25m	52°–58°
			Extended L mode	28m × 25m	10°–22°
RADARSAT-2	24	5.55 cm	Ultra-fine mode	3m × 3m	30°–49°
			Multi-look fine mode	8m × 8m	30°–50°
			Fine mode	8m × 8m	30°–50°
			Standard mode	26m × 25m	20°–49°
			Wide mode	26m × 30m	20°–45°
			*SC narrow mode	50m × 50m	20°–46°
			*SC wide mode	100m × 100m	20°–49°
			Extended H mode	26m × 18m	49°–60°
			*Fine QP mode	8m × 12m	20°–41°
			*Standard QP mode	8m × 25m	20°–41°
Seasat	3	23.44 cm	25m × 25m		20°–26°
SIR-C/X-SAR		23.5cm 5.8cm 3.1cm	(10–50)m - variable		(20°–65°) - variable
TerraSAR-X	11	3.125cm	*SL mode	2m × (1.5–3.5)m	20°–55°
			*HR SL mode	1m × (1.5–3.5)m	20°–55°
			*SM mode	3m × (3–6)m	20°–45°
			*SC mode	16m × 16m	20°–45°

* PL – polarimetric; SC – ScanSAR; SL – spotlight; SM – stripmap; AP - alternating polarization; WS - wide swath; GM - global monitoring; QP = quad polarization; HR – high resolution;

** The azimuth resolution of 10 m is for two looks.

# Resolution (azimuth × ground range) for image mode single look complex images is 6 m × 9 m; for alternating polarization single look complex images is 12 m × 9 m.
